# Effects of time-restricted feeding and type of food on fertility competence in female mice

**DOI:** 10.1038/s41598-022-11251-3

**Published:** 2022-04-29

**Authors:** Nafuko Konishi, Hiroshi Matsumoto, Shu Hashimoto, Udayanga Sanath Kankanam Gamage, Daisuke Tachibana, Aisaku Fukuda, Yoshiharu Morimoto, Masayasu Koyama

**Affiliations:** 1 Women’s Lifecare Medicine, Obstetrics and Gynecology, School of Medicine, Osaka City University, Osaka, 545-8585 Japan; 2 Reproductive Science, Graduate School of Medicine, Osaka City University, 1-4-3 Asahimachi, Abeno-ku, Osaka, 545-8585 Japan; 3 IVF Osaka Clinic, Higashiosaka, Osaka 577-0012 Japan; 4 HORAC Grand Front Osaka Clinic, Osaka, 530-0011 Japan

**Keywords:** Embryology, Endocrine system and metabolic diseases, Reproductive disorders

## Abstract

We assessed the effects of feeding regimen (ad libitum vs. time-restricted food access) and type of food (normal chow (NC: 12% fat) vs. moderately high calorie diet (mHCD: 31% fat)) on fertility competence of female mice. Mice fed mHCD had higher number of oocytes than mice fed NC. On the other hand, when mice were fed NC under time-restricted access to food (NT), the developmental rate to the blastocyst per number of normally fertilized ova was significantly decreased compared to others. The reactive oxygen species (ROS) level in oocytes increased in time-restricted food access and NC group. Transcriptome analysis of whole ovarian tissues from these mice showed a change in the cholesterol metabolism among the four groups. Time-restricted food access decreased serum LDL cholesterol level in both NC and mHCD groups. Moreover, the number of atretic follicles increased in NT mice compared to ad libitum food access mice. The present study shows that mHCD feeding increases the number of ovulated oocytes and that time-restricted feeding of NC impairs the developmental competence of oocytes after fertilization, probably due to the changes in serum cholesterol levels and an increase in the ROS content in oocytes.

## Introduction

Our current lifestyle has led us to eating at inappropriate times/irregular hours and erratic patterns of eating^1^ increasing the risk of chronic diseases, including cancer, cardiovascular diseases, infertility^2^, and other related chronic conditions. Shifting light–dark cycles curtails fertility competence in mice^3–6^. Recently, dietary strategies that focus on the timing of eating and duration of fasting (i.e., chrono-nutrition) have been shown to improve metabolic health in humans^7,8^. Specifically, time-restricted feeding (TRF) limiting food access within the active phase is a dietary strategy that has emerged as a practical intervention for improving insulin resistance along with other markers of whole-body health^1,7–11^. However, there is a question whether chrono-nutrition also affects the fertility competence in females (animals or human) especially oocyte quality and quantity.

Our energy metabolic system has evolved to be cyclical to fit in the daily cycles of food availability. The cyclic change of metabolism is generated from cell-autonomous circadian rhythms and feeding-fasting cycles^12^. A 24-h cycle of this feedback loop is the basis of circadian rhythms in many organisms. The suprachiasmatic nucleus (SCN) is the master regulator of circadian rhythms and is principally adjusted by the light–dark cycle^13^. This feedback loop is also found in peripheral tissues, including the liver, skeletal muscle, and adipose tissue but this loop is not directly operated by light unlike the SCN loop^14–16^. Peripheral clocks are sensitive to fasting-feeding cycles which are driven by Insulin/IGF1^17,18^ and can go out of synchronization with the central clock due to modifications in meal delivery^19^.

A high-fat diet (HFD, > 60% fat) has been shown to be one of the causes of diseases associated with adult lifestyle habits in mice^20^ and HFD resulted in a rapid weight gain and doubling of the body weight. The excessive accumulation of fat has also been involved in damaging female germ cells^21^. The question has also been raised as to whether experimental use of HFD with extremely high levels of fat adequately models the situation of human obesity because the typical western diet contains about 36–40% fat by energy^22^. Given the adverse effects of excessive accumulation of fat on fertility, HFD is not suitable for studies to evaluate the effect of TRF regimen on reproduction.

Here, we subjected inbred mice to either a diet of standard composition (12% fat) or one with moderately high-calorie content (mHCD, 31% fat) under two food-access paradigms: ad libitum (ad lib) or time-restricted access to food (TRF), for more than 11 weeks to assess the effect of time-restricted access on the oocyte quality and quantity of female mice.

## Results

### TRF and mHCD affect the number of follicles

**Figure 1:**
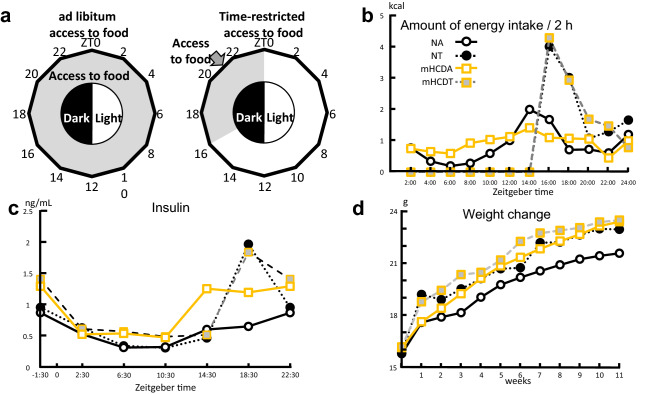
Feeding conditions and effects of TRF or mHCD regimen on energy intake, serum insulin levels, and body weight gain. (**a**) 6-week-old female C57BL/6J mice were housed in groups under a 12 h light:12 h dark schedule for 11 weeks to adapt to the housing condition. They were fed normal chow (NC: 339.4 kcal/100 g) or moderately high-calorie diet (mHCD: 409 kcal/100 g) either with unrestricted (ad libitum: ad lib) or time restricted access to food (TRF). Under TRF, mice were allowed access to food between Zeitgeber time 16 (ZT16, 4 h after lights off) and ZT0 (the time the lights are switched on). Food access was regulated by feeding mice at ZT16 and removing the remaining food at ZT0 daily. Daily energy intake was measured by monitoring the weight of the remaining food at ZT0. Weekly body weight gain was measured at ZT0 once a week. Feeding a normal chow without feeding time limits is a common way of rearing mice. (**b**) The x-axis shows the median value of the measurement time and the y-axis shows the amount of calorie intake per 2 h (kcal). Changes in energy intake of 5 mice/cage were measured every 2 h for 24 h. This measurement was repeated four times. Mice fed NC under an ad lib regimen (NA) displayed diurnal rhythms and nocturnal increase in their energy intake at ZT14. On the other hand, mice fed mHCD under an ad lib regimen (mHCDA) showed dampened diurnal rhythms in energy intake. Because of feeding initiation at ZT16, mice fed NC or mHCD under a TRF regimen (NT and mHCDT) exhibited at least 2 h delay in increase in energy intake compared to their ad lib-fed counterparts. (**c**) The x-axis shows ZT and the y-axis shows the serum insulin levels (ng/mL). NA and mHCDA displayed a clear nocturnal increase in insulin levels. In contrast, due to the initiation of feeding at ZT16, NT and mHCDT exhibited at least 2 h delay in insulin level increase compared to their ad lib-fed counterparts and displayed a rapid increase in insulin levels at ZT18.5. In addition, the insulin levels in TRF regimen sharply rose due to an abrupt increase in energy intake. Data were obtained from 3 replicates at each time for each group. (**d**) The x-axis shows breeding period, and the y-axis shows the body weight. The body weights of mice in NT (n = 35), mHCDA (n = 35), and mHCDT (n = 34) groups increased compared to those in NA group (n = 36, P < 0.01, Supplementary Table 1).

To test whether TRF or mHCD regimen affect the energy intake and body weight gain of female mice, we subjected 6-week-old female mice to normal chow (NC, 12% energy from fat) or mHCD (31% from fat) for 11 weeks under either ad lib or time-restricted access to food during their late nocturnal feeding time (Zeitgeber time 16 (ZT16)-24, Fig. 1a). Mice fed NC under an ad lib regimen (NA) displayed diurnal rhythms in their food absorption (Fig. 1b), exhibiting nocturnal increase in energy intake and serum insulin levels (Fig. 1c). Feeding a normal chow without feeding time limits is a common way of rearing mice. Mice fed mHCD under an ad lib regimen (mHCDA) also displayed a clear nocturnal increase in insulin levels despite dampened diurnal rhythms in energy intake. In contrast, due to the initiation of feeding at ZT 16, mice fed NC or mHCD under TRF regimen (NT and mHCDT) exhibited at least 2 h delay in increases in both energy intake and insulin levels compared to their ad lib-fed counterparts. The insulin levels in TRF regimen sharply rose at ZT18.5 (Fig. 1c) due to an abrupt increase in energy intake at ZT16 (Fig. 1b).

Although there was no difference in the integrated energy intake among the groups (822–873 kcal), TRF increased the body weight of female mice in NC (Fig. 1d) contrary to the previous data in which TRF of HFD (> 60% fat) attenuated body weight gain in male mice without changing energy intake^20^.

**Figure 2:**
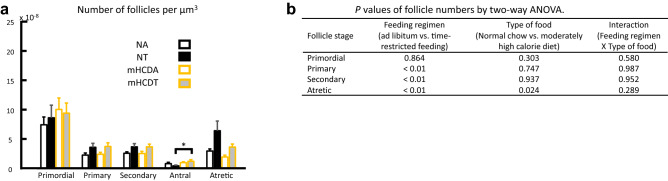
Effects of TRF or mHCD regimen on the number of follicles of female mice (**a**). The data were analyzed by two-way ANOVA following the confirmation of normal distribution. (**b**) The model included the main effects of feeding regimen (ad lib vs. TRF) and type of food (NC vs. mHCD) and their interaction. The number of atretic follicles increased due to TRF (P < 0.01) and decreased due to mHCD (P < 0.05).Meanwhile, the number of primary and secondary follicles increased due to TRF (P < 0.01). The number of antral follicles increased in mHCDT compared with NT by Steel–Dwass test (*P* < 0.05, **a**). *NA* mice fed NC under ad lib regimen, *NT* mice fed NC under time-restricted food access, *mHCDA* mice fed mHCD under ad lib regimen, *mHCDT* mice fed mHCD under time-restricted food access. Data were obtained from ovarian sections of 15 mice in each group. Data were shown mean ± SD. *P < 0.05.

The two-way analysis of variance (ANOVA) showed that the number of primary and secondary follicles increased in both the TRF groups (*P* < 0.01, Fig. 2). The number of antral follicles in the mHCDT increased compared with that in the NT by Steel–Dwass test (*P* < 0.05, Fig. 2).

### mHCD increased the number of ovulated oocytes and TRF constrained embryo development in normal chow

**Figure 3:**
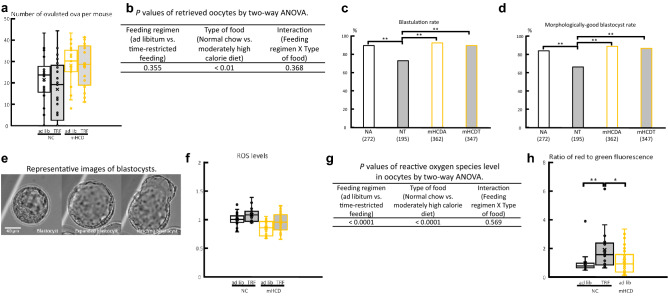
Moderately high calorie diet increased the number of ovulated oocytes, while time-restricted feeding constrained embryonic development and increased reactive oxygen species (ROS) in oocytes and mitochondrial function in oocytes in normal chow. (**a**) The number of ovulated oocytes per mouse increased due to mHCD (P < 0.01) by a two-way ANOVA as shown in (**b**). *ad lib* ad libitum feeding, *TRF* time-restricted feeding, *NC* normal chow, *mHCD* moderately high calorie diet. *NA* mice fed NC under ad lib regimen, *NT* mice fed NC under time-restricted food access, *mHCDA* mice fed mHCD under ad lib regimen, *mHCDT* mice fed mHCD under time-restricted food access. Data were obtained from 22 (NA), 21 (NT), 19 (mHCDA), and 20 (mHCDT) mice. The rates of blastocyst formation (**c**) and morphologically-good blastocysts (**d**) per fertilized ova in NT were significantly lower than others (P < 0.001) by chi-square tests with Bonferroni corrections of the P values. The value in parentheses was number of fertilized ova examined. (**e**) Representative images of blastocysts. Expanded, hatching, and hatched stage blastocysts were defined as morphologically good blastocysts. (**f**) ROS levels were significantly increased due to TRF or NC diet (**g**) (P < 0.01) by two-way ANOVA. (**h**) The ratio of red to green fluorescence (mitochondrial activity) in oocytes obtained from NT group was significantly higher than those of NA and mHCDA groups (P < 0.05) by Steel–Dwass test. Each value was divided by the average value of NA oocytes in the same experiment **(f**,**h**). The data were obtained from 3 independent experiments. The number of oocytes examined were 36 (NA), 27 (NT), 30 (mHCDA), and 35 (mHCDT), respectively in (**f**) and 19 (NA), 17 (NT), and 40 (mHCDA), respectively in (**h**). Data were shown mean ± SD. *P < 0.05 and **P < 0.01.

Do TRF or mHCD regimen affect the fertility competence of female mice? To address this, we subjected the female mice kept for 11 weeks under either ad lib or time-restricted access to food of NC or mHCD to super ovulation treatment and in vitro fertilization. The two-way ANOVA revealed that mice fed mHCD had greater number of ovulated and morphologically-normal oocytes than mice fed NC (*P* < 0.01, Fig. 3a,b, Supplementary Fig. 1). On the other hand, when mice were fed NC under time-restricted access to food from ZT16 to ZT24, the developmental rates to the blastocyst and morphologically-good blastocyst per number of normally fertilized ova significantly decreased (*P* < 0.0001) compared to others (Fig. 3c–e) by chi-square tests with Bonferroni corrections of the *P* values. Ova with 2 pronuclei and 2 polar bodies were defined as normally-fertilized ova.

To assess whether TRF or mHCD regimen affect the oocyte quality, we examined the reactive oxygen species (ROS) content and mitochondrial activity in oocytes. TRF and NC significantly increased the ROS levels in oocytes by two-way ANOVA (*P* < 0.01, Fig. 3f,g). The mitochondrial activity (ratio of red to green fluorescence) was also significantly higher (*P* < 0.05) in oocytes obtained from the NT group than those of other two groups by Steel–Dwass test (NA and mHCDA groups, Fig. 3h).

### TRF delayed circadian rhythm and increased the amplitude of circadian rhythm in liver, fat and ovary

**Figure 4:**
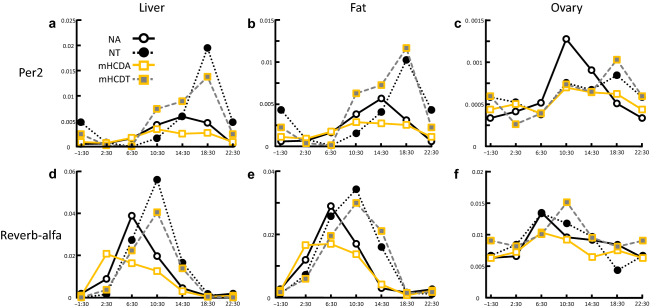
Time-restricted feeding delayed circadian rhythm and increased the amplitude of circadian rhythm. Changes in expression level of Period 2 (Per2) over time in the liver (**a**), fat (**b**), and ovaries (**c**), and Reverb-alfa gene over time in the liver (**d**), fat (**e**), and ovaries (**f**) were seen. The x-axis shows Zeitgeber time while the y-axis shows the ratio of the expression level of each gene per actin. The circadian rhythm (Per2 and Reverb-alfa) in time-restricted feeding groups was delayed compared to their ad lib counterparts in the liver, fat and ovaries. The amplitude of circadian genes was increased by time-restricted feeding in the liver and fat. Data were obtained from 3 replicates at each time for each group. *NA* mice fed NC under ad lib regimen, *NT* mice fed NC under time-restricted food access, *mHCDA* mice fed mHCD under ad lib regimen, *mHCDT* mice fed mHCD under time-restricted food access.

Insulin has been shown to be a liver clock synchronizer^17,18^. In TRF groups, the rise in insulin was delayed by 4 h because of delayed feeding time and insulin levels sharply increased along with the steep energy intake (Fig. 1b,c). Do the delaying of feeding timing and the rapid increase in energy intake affect the peripheral clocks, especially in the ovaries? To answer this, we assessed clock gene expression (Per2 and Reverb-α) in the liver, fat, and ovaries as PER2 is noted for its major role in circadian rhythms^23,24^ and Reverb-α regulates several physiological processes under circadian control, including metabolic and immune pathways^25,26^. The peak time of expression of Per2 and Reverb-α genes in the liver, fat, and ovaries was delayed by 4–8 h when female mice were kept under time-restricted access to food (Fig. 4), showing the retardation of peripheral clocks due to the time-restricted access to food during their late active phase (ZT16-24). Meanwhile, a surge in insulin levels increased the amplitude of circadian genes (Per2 and Reverb-α) expression in the liver and fat, but not in the ovaries (Fig. 4).

### Feeding regimen changed gene expression pattern in ovarian tissues

**Figure 5:**
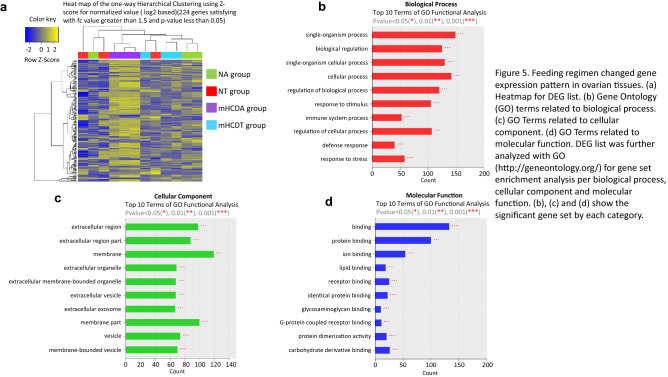
Feeding regimen changed gene expression pattern in ovarian tissues. (**a**) Heatmap for DEG list. (**b**) Gene Ontology (GO) terms related to biological process. (**c**) GO Terms related to cellular component. (**d**) GO Terms related to molecular function. DEG list was further analyzed with GO (http://geneontology.org/) for gene set enrichment analysis per biological process, cellular component and molecular function. (**b**–**d**) The significant gene set by each category.

How do TRF or mHCD regimen affect the fertility competence of female mice? To explore this, we performed transcriptome analysis of ovaries of mice kept for 11 weeks under either ad lib or time-restricted access to food of NC or mHCD. The transcriptome analysis showed that 224 genes differentially expressed among the 4 groups in ovarian tissues as shown in the heat map of the one-way hierarchical clustering (Fig. 5a). The DEG list was further analyzed with Gene Ontology for gene set enrichment analysis per biological process, cellular component and molecular function (Fig. 5b–d). For listing significant genes, gene-set enrichment analysis was performed based on KEGG database. KEGG pathway viewer provided the pathway by fold change for genes that were differentially and significantly expressed in each comparison pair using pathway map information of Mus musculus (Table 1). Transcriptome data were shown in Supplementary Table 2.

**Table 1:** Pathways by fold change for significantly expressed genes by each comparison pair using pathway map information of Mus musculus in ovarian tissues.

Systems	Sub-systems	Pathways	Significantly expressed genes among treatment	mHCA vs. mHCT	mHCA vs. NA	NA vs. NT	mHCA vs. NT	mHCT vs. NT
Metabolism	Global and overview maps	Metabolic pathways mmu01100	**P < 0.001**	P > 0.05	**P < 0.05**		**P < 0.01**	
	Carbohydrate metabolism	Amino sugar and nucleotide sugar metabolism mmu00520	**P < 0.01**				**P < 0.01**	
	Lipid metabolism	Steroid hormone biosynthesis mmu00140	**P < 0.05**	P > 0.05		P > 0.05		P > 0.05
		Arachidonic acid metabolism mmu00590	**P < 0.05**		P > 0.05		P > 0.05	
	Glycan biosynthesis and metabolism	Glycosaminoglycan biosynthesis—keratan sulfate mmu00533	**P < 0.05**	**P < 0.05**	P > 0.05			
Environmental Information Processing	Signal transduction	Hippo signaling pathway mmu04390	**P < 0.01**	P > 0.05	P > 0.05	P > 0.05	P > 0.05	
		NF-kappa B signaling pathway mmu04064	**P < 0.01**		P > 0.05		**P < 0.01**	
	Signaling molecules and interaction	Cytokine-cytokine receptor interaction mmu04060	**P < 0.001**	P > 0.05	P > 0.05		**P < 0.01**	P > 0.05
		Cell adhesion molecules (CAMs) mmu04514	**P < 0.001**	P > 0.05	P > 0.05		P > 0.05	
Cellular Processes	Transport and catabolism	Phagosome mmu04145 Lysosome mmu04142	**P < 0.001** **P < 0.05**	P > 0.05 P > 0.05	**P < 0.001** **P < 0.01**		**P < 0.001** **P < 0.05**	
	Cell growth and death	Apoptosis mmu04210	**P < 0.01**				**P < 0.001**	
	Cellular community—eukaryotes	Tight junction mmu04530	**P < 0.01**	**P < 0.01**			P > 0.05	
Organismal Systems	Immune system	Hematopoietic cell lineage mmu04640	**P < 0.01**	P > 0.05	P > 0.05		**P < 0.01**	
		Complement and coagulation cascades mmu04610	**P < 0.001**		**P < 0.01**		**P < 0.001**	
		Platelet activation mmu04611	**P < 0.05**		P > 0.05		P > 0.05	
		C-type lectin receptor signaling pathway mmu04625	**P < 0.01**	P > 0.05	P > 0.05		P > 0.05	
		Natural killer cell mediated cytotoxicity mmu04650	**P < 0.01**		P > 0.05		**P < 0.001**	
		IL-17 signaling pathway mmu04657	**P < 0.05**	P > 0.05	P > 0.05			P > 0.05
		Leukocyte transendothelial migration mmu04670	**P < 0.001**	P > 0.05	P > 0.05	P > 0.05	**P < 0.001**	
		Intestinal immune network for IgA production mmu04672			**P < 0.05**		P > 0.05	
		Chemokine signaling pathway mmu04062	**P < 0.001**	P > 0.05	P > 0.05		P < 0.05	P > 0.05
	Endocrine system	PPAR signaling pathway mmu03320	**P < 0.01**		P > 0.05		**P < 0.001**	
		Renin-angiotensin system mmu04614	**P < 0.001**	**P < 0.001**			**P < 0.01**	
	Digestive system	Cholesterol metabolism mmu04979	**P < 0.001**	P > 0.05	P > 0.05		**P < 0.001**	
	Development	Osteoclast differentiation mmu04380	**P < 0.001**		**P < 0.001**		**P < 0.001**	
Human Diseases	Cancers: overview	Pathways in cancer mmu05200	**P < 0.01**	P > 0.05	**P < 0.05**	P > 0.05	P > 0.05	
	Cancers: specific types	Acute myeloid leukemia mmu05221	**P < 0.05**				**P < 0.01**	
	Immune diseases	Asthma mmu05310	P > 0.05		P > 0.05		**P < 0.05**	
		Systemic lupus erythematosus mmu05322	**P < 0.001**		P > 0.05		**P < 0.001**	
		Rheumatoid arthritis mmu05323	**P < 0.001**	P > 0.05	**P < 0.001**		**P < 0.001**	
	Neurodegenerative diseases	Prion diseases mmu05020	**P < 0.05**				**P < 0.05**	
	Infectious diseases: bacterial	Pertussis mmu05133	**P < 0.001**		P > 0.05		**P < 0.001**	
		Staphylococcus aureus infection mmu05150	**P < 0.001**		**P < 0.05**		**P < 0.001**	
		Tuberculosis mmu05152	**P < 0.001**	P > 0.05	**P < 0.001**		**P < 0.001**	P > 0.05
	Infectious diseases: parasitic	Leishmaniasis mmu05140	**P < 0.001**	P < 0.05			**P < 0.001**	
		Chagas disease (American trypanosomiasis) mmu05142	**P < 0.01**		P > 0.05		**P < 0.01**	

### Effect of feeding time and mHCD on serum lipid levels

**Figure 6:**
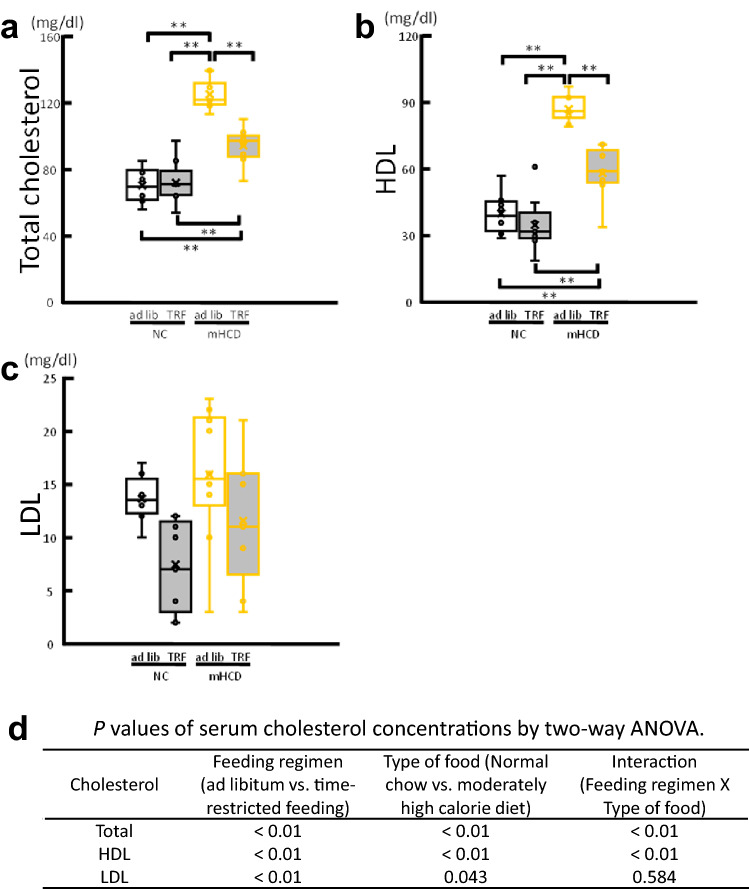
Feeding regimen changed serum lipid levels. The total (**a**), high-density lipoprotein (HDL, **b**), and low-density lipoprotein (LDL, **c**) cholesterol in the serum was measured. Analysis by Tukey Kramer test following ANOVA showed higher levels of total and HDL cholesterol in mice fed mHCD than mice fed NC (**a**,**b**). The analysis by two-way ANOVA revealed that time restricted regimen decreased the LDL levels (P < 0.01, **c**). Moreover, mHCD increased LDL levels (P < 0.05, **c**). Data were obtained from 8 (NA), 9 (NT), 10 (mHCDA), and 9 (mHCDT) mice. The data of cholesterols were analyzed by two-way ANOVA. The model included the main effects of feeding regimen (ad lib vs. TRF) and type of food (NC vs. mHCD) and their interaction. When the interaction was significant, the data were analyzed by Tukey Kramer test following ANOVA (**a**,**b**). **P < 0.01.

**Figure 7:**
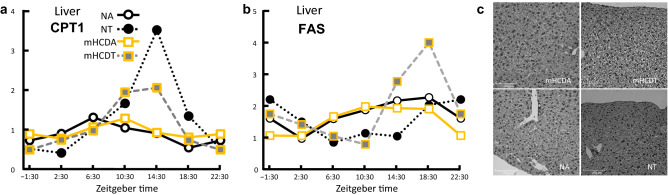
Feeding regimen affected fat degradation and synthesis in liver. (**a**) Changes of carnitine palmitoyltransferase 1 (CPT1) gene expression in liver over time. CPT1 is the rate-limiting enzyme in fatty acid metabolism. A peak in CPT1 gene expression was observed before the increase in energy intake. The peak of CPT1 gene expression in NT mice was remarkably high among the 4 groups. (**b**) Changes in fatty acid synthase (FAS) gene expression in liver over time. FAS catalyzes fatty acid synthesis. A peak in FAS gene expression was observed after an increase in energy intake. Data were obtained from 3 replicates at each time for each group. (**c**) Representative images of HE sections of livers were shown from 4 groups. There were no signs of fatty liver in any of the groups.

Transcriptome analysis of ovarian tissues showed that metabolic pathways (mmu01100), steroid hormone biosynthesis (mmu00140), PPAR signaling pathway (mmu03320), glycosaminoglycan biosynthesis-keratan sulfate pathway (mmu00533), cholesterol metabolism (mmu04979) including apolipoproteins B, C, and E, and lipoprotein lipase, and phospholipid transfer protein were significantly changed (Table 1) in their gene expression pattern. Accordingly, to see how TRF or mHCD regimen affects the serum lipid levels in female mice, the total, high-density lipoprotein (HDL), and low-density lipoprotein (LDL) cholesterol in the serum were measured. Mice fed mHCD under both the feeding regimen showed higher levels of total and HDL cholesterol than mice fed NC under both the feeding regimens (Fig. 6a,b) when the data were analyzed by Tukey Kramer test following ANOVA. The two-way ANOVA revealed that the LDL cholesterol was significantly decreased by TRF regimen (P < 0.01, Fig. 6c,d) and increased by mHCD (*P* < 0.05). Lowered levels of LDL cholesterol under TRF regimen suggest a shortage of lipid supply in NT due to decreased lipoprotein levels. Lipoproteins are responsible for transporting lipids (fats) around the body in the extracellular fluid, making fats available to body cells for receptor-mediated endocytosis^27,28^. Next, we assessed the temporal alteration in gene expression of carnitine palmitoyltransferase 1 (CPT1) and fatty acid synthase (FAS) in the liver, the key enzymes of fatty acid metabolism and synthesis, respectively. Under an ad lib regimen, the expression of CPT1 gene reached a peak just prior to or around the start of the active phase (ZT 6:30 for NA, ZT 10:30 for mHCDA, Fig. 7a). However, in the TRF mice, the CPT1 expression soared at ZT 14:30, immediately before access to the food. The expression of CPT1 gene probably reflected increased fat catabolism before the starting time of feeding (Fig. 1b). The expression of FAS gene increased after feeding (Fig. 7b). Its rapid increase was not observed in NT compared to mHCDT group possibly due to the lower serum cholesterol levels. Although the perirenal adipose mass in mHCD group (mHCDA: 0.212 g, mHCDT: 0.147 g) increased compared to NC group (*P* < 0.0001, NA: 0.104 g, NT: 0.111 g), no lipid droplets, an indicator of hepatic steatosis, were seen in liver slices from any of the groups (Fig. 7c).

### TRF increased the atretic follicles in normal chow

**Figure 8:**
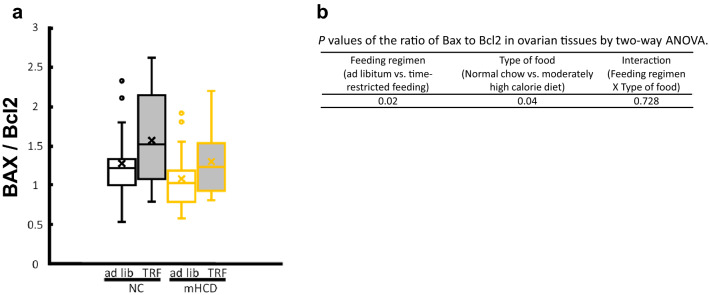
Time-restricted feeding and NC increased the ratio of Bax to Bcl2 in ovaries by a two-way ANOVA (P < 0.05, **a**). (**b**) The model included the main effects of feeding regimen (ad lib vs. TRF) and type of food (NC vs. mHCD) and their interaction. Data were obtained from 18 mice in each experimental group.

Transcriptome analysis also indicated a statistical change in the cytokine-cytokine receptor interaction (mmu04060), renin-angiotensin system (mmu04614), lysosome (mmu04142), phagosome (mmu04145), and apoptosis (mmu04210) including pro-survival gene (hematopoietic Bcl-2-related protein A1) (Table 1). Recently, the presence of a concerted mechanism involving the complement system to allow simultaneous growth and degeneration of the different follicular compartments in a timely manner has been suggested^29^. Thus, we examined the expression levels of apoptosis-related genes (Bax and Bcl2) using qPCR and calculated the ratio of Bax to Bcl2. The feeding regimen or type of food significantly affected (*P* < 0.05) the Bax to Bcl2 ratio Fig. 8) and the number of atretic follicles in the ovaries (Fig. 2).

### Effect of feeding time and mHCD on proinflammatory cytokines

Complement and coagulation cascades (mmu04610), leukocyte trans-endothelial migration (mmu04670), systemic lupus erythematosus (mmu05322), rheumatoid arthritis (mmu05323), cell adhesion molecules (CAMs) (mmu04514), and antigen processing and presentation (mmu04612) were shown to be changed in this study (Table 1). Moreover, transcriptome analysis indicated a change in steroid hormone biosynthesis (mmu00140) due to downregulation of corticosteroid 11-beta-dehydrogenase isozyme 2 (Hsd11b2) and 20alpha-hydroxysteroid dehydrogenase (Akr1c18) in NT mice compared to others, leading to a possible decrease in the pregnane (21-carbon) steroid hormone. Based on these transcriptome analyses, we measured the changes in inflammatory cytokines. Most of them fell below the lower limit of quantification (LLOQ) even when undiluted serum was measured. Therefore, we compared cytokine levels between NA and NT using median fluorescent intensity (MFI) and found that TRF delayed the time course of serum cytokine levels (INFγ, IL2 and IL10) by 4 h (Supplementary Fig. 2).

## Discussion

The results of the present study showed that mHCD feeding increases the number of ovulated oocytes and morphologically normal oocytes per mouse in both feeding regimens (ad-lib and time restricted) and that TRF of NC impairs the developmental competence of oocytes after in vitro fertilization.

mHCD feeding increased the levels of total, HDL, and LDL cholesterol (Fig. 6a–c). According to the conventional interpretation, higher levels of total and LDL cholesterol increase the risk of cardiovascular diseases, as LDL cholesterol invades the endothelium where it gets oxidized. However, this issue remains controversial and is not true for lower levels of LDL^30,31^. The natural steroid hormones including estradiol and progesterone are generally synthesized from cholesterol in the gonads and adrenal glands^32^. In addition, positive correlation has been shown between total or LDL cholesterol and anti-Müllerian hormone levels, a marker of ovarian reserve^33^. Accordingly, lower levels of total and LDL cholesterol lead to a possible decrease in steroid hormone levels, and in turn a decrease in the number of retrieved oocytes when NC was fed, especially in TRF, perhaps due to an increase in fat degradation (Fig. 7a; Hatori et al.^20^). Meanwhile, membrane cholesterol levels have been shown to influence membrane permeability and stability, and plasma membrane protein activity^34^. Moreover, LDL has been shown to bind to bacteria and endotoxins and neutralize them before they can negatively affect the host cells^35,36^. From these essential roles of LDL, we can deduce that a significantly low level of LDL in NT mice could lead to a decrease in oocyte developmental competence due to an increase in intracellular ROS levels. Although higher LDL levels are believed to be detrimental to overall body health, LDL is an indispensable component of the cellular membrane. Thus, a serious shortage of LDL could cause various cell dysfunctions, including ovarian and oocyte incompetence. Hence, mice fed mHCD could yield more oocytes than NC. Here, we postulate that though controlled lipid intake is essential to maintain the female fertility status, a strict limitation of diet could be detrimental to reproductive health.

Unlike previous data, which showed a doubling of body weight, our study, although statistically significant, showed only a 6–9% increase in body weight (Fig. 1d). As noted in introduction, the excessive accumulation of fat has also been involved in damaging female germ cells^21^. Given the adverse effects of excessive accumulation of fat on fertility, the mHCD diet was used. The results showed that despite similar body weights, the reproductive performance of mice fed the mHCD diet was higher than that of the NT group. This could be attributed to the amount of cholesterol in the blood and the amount of ROS in the oocytes, but the differences between mHCD and NT were not completely determined. The mechanism that caused this has yet to be explained.

TRF has been widely reported to not only extend life span but also protect against various pathological conditions of mice fed high fat diet (HFD, > 60% fat, Hatori et al.^20^). Based on the previous data^35,36^ we expected an improvement in the fertility of mice especially in mHCD group by TRF. Recently, it has also been shown that TRF increases follicle number and litter size, and decreases the period required for pregnancy in both NC and HFD^37^. However, contrary to our expectations, TRF in the present study constrained embryonic development in NC (Fig. 3c,d). The positive effects of TRF, such as an acceleration of fat metabolism, decrease in fat store, and suppression of fat accumulation in the liver has been shown in HFD-induced obesity and related metabolic disorders but not in TRF of NC (< 15% fat^20,38^). Under ad lib condition mice fed NC were observed to be eating during dark phase and resting during light phase (Fig. 1b) similar to the previously reported data^20^. But this was not the case with HFD (> 60% fat) and mHCD (31% fat) fed mice. Metabolic stimulation, including an acceleration of fat metabolism, decreased fat store, and suppression of fat accumulation in the liver due to clear circadian rhythm in the liver that was observed by TRF of HFD^20^ was also observed in the present study and the expression of Per2 and Reverb-alfa in the liver increased in both NC and mHCD under TRF regimen (Fig. 4). Accordingly, the gene expression of CPT1, the rate controlling enzyme of fatty acid metabolism in mitochondria surged just prior to feeding at ZT14.5, especially in NT group in order to compensate for insufficient lipid supply, such as lower cholesterol levels. On the other hand, the expression of FAS gene increased only in mHCD under TRF regimen, not in NC, possibly because of an insufficient accumulation of acetyl-CoA as shown by its low concentration of serum cholesterols. Specifically, the levels of LDL cholesterol were significantly low (Fig. 6c). Thus, a sharp decline in serum LDL could negatively affect oocyte development due to adverse phenomena such as ROS accumulation.

Moreover, transcriptome analysis results suggested a decrease in pregnane (21-carbon) steroid hormone in NT mice compared to others (Table 1). Cortisone is a pregnane (21-carbon) steroid hormone and suppresses various elements of the immune system^39^, thus reducing inflammation and incidental pain and swelling. Meanwhile, there exist risks including toxicity to articular cartilage and numerous systemic side effects such as an increase in blood glucose levels, a reduction in immune function, and an increased risk of infections in the long-term exposure to cortisone^40^. Accordingly, we speculated that there could be inflammatory stress in NT group due to a lack of cortisone synthesis. Although there was no difference in the inflammatory cytokine levels, a 4-h phase shift was observed, possibly due to a corresponding delay in peripheral circadian rhythm in accordance with feeding time. Recently, the concept of circadian immunity has been proposed, in which the circadian behavior of the immune system allows optimization of immune response throughout the circadian cycle^41,42^. A long-term circadian asynchronization between the immune system circadian behavior and the SCN circadian cycle has been shown to exacerbate immune senescence and consequent chronic inflammation^43^. In addition, the ovulation mechanism is similar to inflammatory reaction^44^ and accompanies production of ROS, leading to a decrease in oocyte developmental competence^45^. In this study the TRF regimen increased ROS content in oocytes due to which, the developmental competence decreased in oocytes from mice kept under a TRF regimen. However, the underlying mechanism involved in the relationship between the circadian asynchronization and its deleterious effects on oocyte health needs further study.

Kisspeptin neurons which are located in the hypothalamus as with GnRH neurons are recognized to orchestrate the hypothalamus-pituitary-gonadal (HPG) axis, a robust mechanism for the regulation of reproduction both in male and female mammals^46–48^. As the main regulatory clock, the SCN, is controlled by the light–dark cycle^12^, but the circadian rhythms of peripheral tissues deviate from the SCN cycle due to insulin/IGF1-mediated feeding and fasting cycles^17,18^, our analysis was focused on the displacement of circadian rhythms in peripheral tissues such as ovary. Delezie et al.^49^ have shown that Rev-erbα in the brain is not only required for proper functioning of the light-regulated master clock, but also for integrating feeding cues essential for animal survival and for regulating circadian behavior and physiology in response to environmental conditions. In light of this, the failure to adequately examine changes in the HPG axis may be a flaw in this study. This issue should be addressed in future studies.

When mice were fed NC under TRF regimen from ZT16 to 24, the developmental rate to the blastocyst decreased. To figure out the reasons behind the lower development potential in NT mice, we measured intracellular ROS content and mitochondrial activity in oocytes, where both were found to be increased. The ROS level was as expected but not the mitochondrial activity. It has been shown that both ROS content and mitochondrial activity are higher in murine oocytes with lower developmental competence^50^ although higher mitochondrial activity has been believed to be a marker of oocytes with higher developmental competence^51^. To compensate for the insufficient ATP production in oocytes, the mitochondrial electron transport system in oocytes may become excessively active and result in an accumulation of extra ROS.

Irregular daily eating patterns have been shown to have adverse effects on circadian biology^52^. Restricting food access only at the inactive phase in mice completely reversed the phase of the circadian clock in the liver, stomach, intestine, heart, pancreas, and kidney, without affecting the phase of the circadian clock in the SCN^19,53^. Simply delaying meals by 4 h also resulted in a corresponding phase shift in the circadian clock in mouse liver. This in turn delayed the peaking of several genes involved in glucose homeostasis and transcription factors involved in lipid homoeostasis in the liver^54^. Late feeding also delayed the peak times of insulin, free fatty acids, and bile acids in plasma and the circadian rise in body temperature and increased body weight. This shows that cues from the fasting-feeding cycle are stronger entraining cues for peripheral clocks than the light–dark cycle and therefore, delayed feeding time causes disruption in circadian rhythm of peripheral tissues^19,55^.

It has been shown that when female mice were fed under TRF condition for 8 weeks and then housed with males, they became pregnant and had more pups in a shorter period than when they were fed under ad lib condition^37^. This data does not necessarily contradict our results, where the number of ovulations was the same, but the ability to develop into blastocysts was reduced in the case of NC feeding. The use of NC with a lower fat percentage than Hua et al.^37^ in the present experiment may have had a negative effect on oocyte development. Furthermore, as the feeding time was 8 h in the present study, which was shorter than the 10 h in Hua et al.^37^, cholesterol deficiency may have led to an increase in atretic follicles and a decrease in egg quality. In addition, since Hua et al.^37^ did not specify the time of feeding initiation we cannot compare the results. In our case, feeding was started from 4 h after light-off. As a result, circadian rhythms in the liver, adipose, and ovaries were shifted by about 4 h, and fluctuations in some inflammatory cytokine levels were also observed to be delayed by 4 h. It has been shown that delaying the onset of TRF delays the rise in body temperature by 4 h, resulting in weight gain and increased adipose tissue due to decreased energy expenditure^54^. It has also been suggested that a delay in TRF initiation time causes dysregulation of core body temperature, sleep–wake cycle, and hippocampal gene expression, resulting in impaired formation of long-term memory^56^. Exposure to artificial light during resting phase in humans has been thought to cause possible negative effects on pregnancy mainly due to disruption in central circadian rhythm^2,3^. Hence, the difference between Hua et al.^37^ and our data is most likely due to the difference in feeding initiation time.

Meanwhile, a diet containing higher calories under ad libitum feeding has been shown to drastically change circadian metabolites^57^. Thus, the difference in TRF effect on the blastocyst development in this study could be due to the difference in circadian metabolites between NC and mHCD. Our data turns the spotlight on the possibility that the disturbance of regular circadian rhythm in peripheral tissues could also negatively affect reproduction.

Excessive consumption of calories under normal nutritional conditions has been shown to negatively affect fertility^58^. TRF has been developed to maintain the good health of mice without becoming obese. Meanwhile, TRF has been shown to inhibit calorie accumulation and promote calorie consumption, reducing the risk of lifestyle related diseases^59^. Accordingly, a combination of NC and TRF could result in decreased cholesterol levels and lead to lower oocyte quality.

In conclusion, the present study reveals that a moderately high calorie diet increases the number of oocytes and that TRF with NC increases intracellular ROS level and mitochondrial activity in oocytes, leading to a decrease in the developmental ability of oocytes after insemination. Although TRF has been widely believed to provide beneficial effects on various pathological conditions of HFD-fed mice, TRF altered the fertility competence of female mice probably due to shortage of lipids, such as cholesterols. TRF regimen in NC could be an animal model for infertility due to over exercise, such as in the case of athletes.

Our data offers a new insight into the effects of feeding regimen on fertility competence and that TRF does not always support a healthy life, cautioning us that regardless of how good a therapy may be, it may have negative effects.

## Methods

### Animals

This study was approved by the Institutional Animal Care and Use Committee of Osaka City University (Permission number: 18025). In addition, all experiments involving live animals were performed in accordance with relevant guidelines and regulations and were reported as described by the recommendations in the ARRIVE guidelines. The feeding regimen experiments were repeated in 7 independent batches of mice. All mice were sacrificed by cervical dislocation.

### Feeding schedule and diets

Six-week-old female C57BL/6J mice from Japan SLC Inc. (Hamamatsu, Shizuoka, Japan) were housed in groups (3–5 mice per cage) under a 12 h light:12 h dark schedule for 11 weeks to adapt to the housing condition. They were fed NC (CE-2, Clea Japan, Inc.: 29.9% protein, 11.6% fat, 58.5% carbohydrates; 339.4 kcal/100 g) or mHCD (Quick Fat, Clea Japan, Inc.: 24.6% protein, 30.7% fat, 44.7% carbohydrates; 409 kcal/100 g) either with unrestricted food access (ad lib) or TRF (see Fig. 1). Under TRF, mice were allowed access to food between ZT16 (4 h after lights off) and ZT0 (the time the lights are switched on). Food access was regulated by feeding mice at ZT16 and removing remaining food at ZT0 daily. Daily energy intake was measured by monitoring the weight of the remaining food at ZT0. Weekly body weight gain was measured at ZT0 once a week.

### In vitro fertilization and culture

After the diet management for 11 weeks, a total of 82 mice (NA: 22, NT: 21, mHCDA: 19, mHCDT: 20) were superovulated by 5 IU PMSG administration (Serotropin^®^ ASKA Animal Health Co., Ltd., Tokyo, Japan) followed by 5 IU human chorionic gonadotropin (hCG) administration 48 h later. Ovulated oocytes were retrieved from oviductal ampulla at 14 h post hCG injection and cocultured with sperm retrieved from male mice (9 weeks, C57BL/6J mice from Japan SLC Inc.) in HTF. After 6 h, normally-fertilized ova confirmed by the presence of male and female pronuclei were cultured in KSOMaa^60^ (Ark resource co., Kumamoto, Japan) under 5% CO_2_, 5% 0_2_, and 90% N_2_ with high moisture content. The blastocyst development was assessed at 96 h after in vitro fertilization. Expanded, hatching, and hatched stage blastocysts were defined as morphologically good blastocysts.

### ROS content and mitochondrial function in oocytes

Cumulus-oocyte complexes retrieved from oviducts at 14 h post hCG injection were denuded by pipetting after hyaluronidase treatment. Denuded oocytes were washed in Ca^2+^- and Mg^2+^-free PBS (PBS (−)) supplemented with 0.1% poly (vinyl alcohol) (PVA−PBS) and stained with 1 μM H2DCFDA (D399, Thermo Fisher Scientific, Foster City, CA, USA) PVA-PBS for 10 min at room temperature (RT) under light shielding as previously described^61^. Just before the start of each experiment, fresh H2DCFDA was prepared at 1 μM in PVA−PBS from stock solution (1 mM in DMSO). Stained oocytes were washed in PVA−PBS to remove the traces of the dye and were transferred into a 3-μL droplet of PVA-HTF on a glass-bottomed culture dish (P35G-0-14-C; MatTek Corporation, Ashland, MA, USA). Microscopic images were obtained using a CLM (CellVoyager CV1000; Yokogawa Electronic, Tokyo, Japan) at 40× at RT in air and fluorescence intensity in the equatorial plane of oocytes was measured using ImageJ (http://imagej.nih.gov/ij/). In this study, a total of 128 oocytes from 3 mice in each group were used for the analysis of ROS content in 3 independent runs. The ratio of fluorescence intensity of each sample was divided by the mean intensity of mice which were fed NC under an ad lib regimen (NA group) in each run and was compared.

To assess mitochondrial activity, denuded oocytes were incubated with 10 μg/mL JC10 (Cell Meter™ JC-10 Mitochondrial Membrane Potential Assay Kit; AAT Bioquest, Inc., Sunnyvale, CA, USA) for 0.5 h in bicarbonate-buffered HTF supplemented with 0.1% poly (vinyl alcohol) (PVA-HTF) for 30 min at 39 °C under 5% CO_2_ in air with high moisture content and then washed thrice in PVA-HTF. Stained oocytes were transferred into a 3-μL droplet of PVA-HTF on a glass-bottomed culture dish. Microscopic images were obtained using a CLM at 40× and 39 °C under 5% CO_2_ in air with high moisture content and fluorescence intensity in the equatorial plane of oocytes was measured using ImageJ. In this study, a total of 76 oocytes from 3 mice in each group except for mHCDT were used for the analysis of mitochondrial activity in 3 independent runs. The ratio of red to green fluorescence was calculated as mitochondrial activity. The ratio of each sample was divided by the mean value of NA group in each run and was compared to others.

### Organ and serum collection

Reproductive staging of estrous cycles was evaluated over 11 days following 10 weeks of feeding regimen. Briefly, vaginal smears were performed daily at ZT1 (1 h post the start of the light cycle) to obtain vaginal epithelial cells which were stained with Giemsa’s stain solution (37114-35, Nacalai tesque, Inc., Kyoto, Japan) and evaluated for the relative abundance of leukocytes, nucleated vaginal epithelial cells, and cornified cells using phase-contrast light microscopy. Within each feeding group, 3 mice at diestrus stage were sacrificed every 4 h over a 24-h period. Liver, white adipose tissue (perinephric), and ovary from individual mice were flash frozen in liquid nitrogen or fixed in 4% paraformaldehyde. Frozen samples were used for RNA analyses, while the fixed samples were used for histological examination after dehydrating in alcohol gradients. The serum was also obtained from the vein of lower part of the auricle of mice every 4 h over a 24-h period for measuring insulin and inflammatory cytokines.

### Hepatic histology and ovarian follicle counts

Sections (4 µm) of formalin-fixed liver and ovaries obtained from 15 mice in each group were stained with hematoxylin and eosin (H&E) and observed under phase-contrast light microscopy.

Every three serial ovarian sections at 10 µm interval were assessed to determine follicle numbers as previously described^62^. Only those follicles that contained a visible nucleated oocyte were counted to avoid counting the same follicle more than once. All follicles were characterized based on their morphology. Briefly, primordial follicles consisted of an oocyte surrounded by a single layer of flattened granulosa cells, primary follicles consisted of an oocyte surrounded by a single layer of cuboidal granulosa cells, secondary follicles consisted of an oocyte surrounded by multiple layers of cuboidal granulosa cells with no antrum and antral follicles consisted of an oocyte surrounded by several layers of cuboidal granulosa cells with visible antrum. Atretic follicles were characterized by oocyte and/or granulosa cell degeneration, and/or retraction of the granulosa cell layer from the oocyte, or theca cell layer and nuclear blebbing. Total volume of each section was calculated (area of the section x thickness of the section). Follicle density (μm^3^) was calculated by dividing the number of follicles in each stage of each section by the total volume of each ovarian section calculated by the method described above^63^.

### Insulin

Assessment of serum insulin was performed using Mouse Insulin ELISA kit (Morinaga Institute of Biological Science, Inc. Kanagawa, Japan) following manufacturer’s protocols. The absorbance at 450 nm was measured using the VarioskanLUX (Thermo Fischer Scientific).

### Cholesterol, HDL, LDL

Plasma total, HDL, and LDL cholesterol obtained from female mice at ZT 1:30–2:00 were measured using a Roche cobas b 101 Lipid Control instrument (Roche Diagnostics KK, Tokyo, Japan).

### Inflammatory cytokines

Serum cytokine levels were analyzed by multiplex immunoassays and Luminex bead-based antibody capture and recognition arrays (LEGENDplex™, BioLegend, San Diego, CA). Undiluted samples from mice fed NC under ad lib or TRF were run using a cell sorter (SH800, Sony Corporation, Tokyo, Japan). Data were collected with the LEGENDplex v8.0 Software. IL-2, IL-10, IFNγ, and TNFα were measured.

### RNA Extraction and transcriptome analyses

Total RNA from liver, fat tissue, and ovaries was extracted separately and purified with the Isogen (331-02501, NIPPON GENE CO., LTD., Toyama, Japan) according to the protocol. Transcriptome analyses were performed as described in supplementary methods.

### cDNA synthesis, and quantitative PCR (qPCR)

A fixed amount of total RNA obtained from the liver, fat tissue, and ovaries was reverse-transcribed with QuantiTect^®^ Reverse Transcription Kit (Qiagen) according to the manual. The expression of clock and metabolic-related genes was evaluated by qPCR (Rotor-Gene Q, Qiagen) with the following reaction system: 2 µL of cDNA was incubated with 25 pmol of both reverse and forward primer from gene of interest (see Supplementary Table 3 for primer sequences) and 12.5 µL of SYBR Green master mix (QuantiFast SYBR Green PCR Kit, Qiagen) and was made up to a final volume of 25 µL with RNase free water. In the analysis of expression level of actin and clock genes, a standard curve was generated in each assay by tenfold serial dilution of the cDNA samples including PCR amplification target region (Supplementary Table 4). The relative amount of each gene was normalized against the mean of actin. Analysis of the genes associated with metabolism and apoptosis was performed by the delta delta CT method and relative expression levels were calculated in comparison to actin as a control.

### Statistical analysis

The number of follicles at various stages except for antral follicles, ovulated and morphologically normal oocytes per mouse, cholesterol levels, ratio of BAX per Bcl2, and ROS levels were compared by two-way ANOVA after confirming the normal distribution by Kolmogorov–Smirnov test following logarithmic transformation. The model included the main effects of feeding regimen (ad lib vs. TRF) and type of food (NC vs. mHCD) and their interaction. When the interaction was significant, the data were analyzed by a Tukey–Kramer test following ANOVA. The integrated energy intake after 11 weeks, body weight over 11 weeks, and mitochondrial activity in oocytes were compared by a Tukey–Kramer test following ANOVA. Nonparametric test (Steel–Dwass) was performed if the normal distribution was not confirmed. Blastulation rates per fertilized ova were compared by chi-square tests with Bonferroni corrections of the *P* values. *P* values less than 0.05 were considered significant. Data were shown as mean ± SD. Statistical analyses were performed using EZR software^64^.

## Supplementary Information

Supplementary Information 1.

Supplementary Information 2.

Supplementary Information 3.

Supplementary Information 4.

## Data Availability

The data that support the findings of this study are available from the corresponding author.
