# Supplementary material for: Effects of time-restricted feeding and type of food on fertility competence in female mice
Source: Sci Rep. 2022 Apr 29;12:7064. doi: 10.1038/s41598-022-11251-3 (PMC9054750; doi:10.1038/s41598-022-11251-3)
Supplement: Supplementary file 2 — Supplementary Information 2. [file 41598_2022_11251_MOESM2_ESM.pptx]

## Slide 1
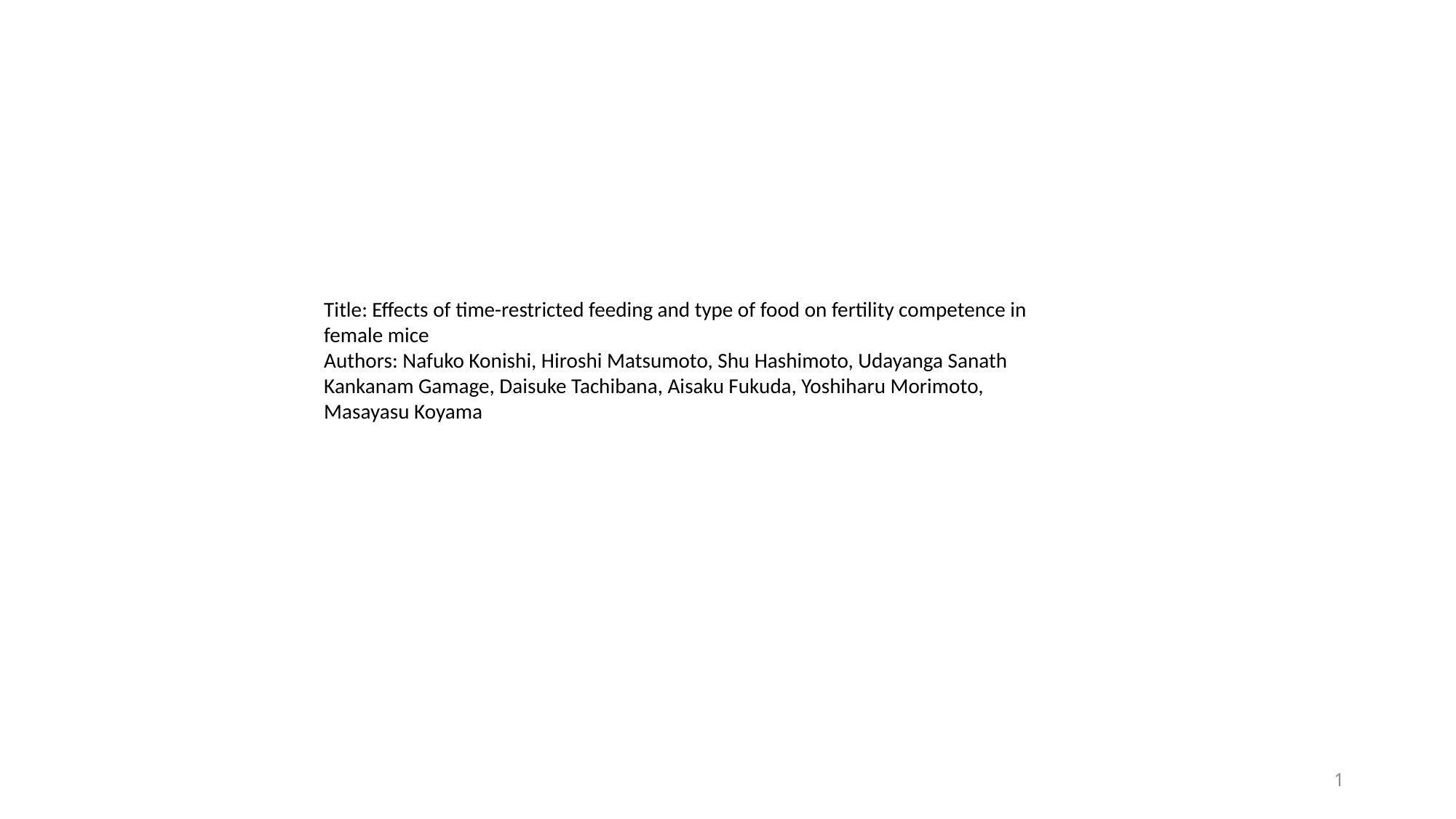

Title: Effects of time-restricted feeding and type of food on fertility competence in female mice
Authors: Nafuko Konishi, Hiroshi Matsumoto, Shu Hashimoto, Udayanga Sanath Kankanam Gamage, Daisuke Tachibana, Aisaku Fukuda, Yoshiharu Morimoto, Masayasu Koyama
1

## Slide 2
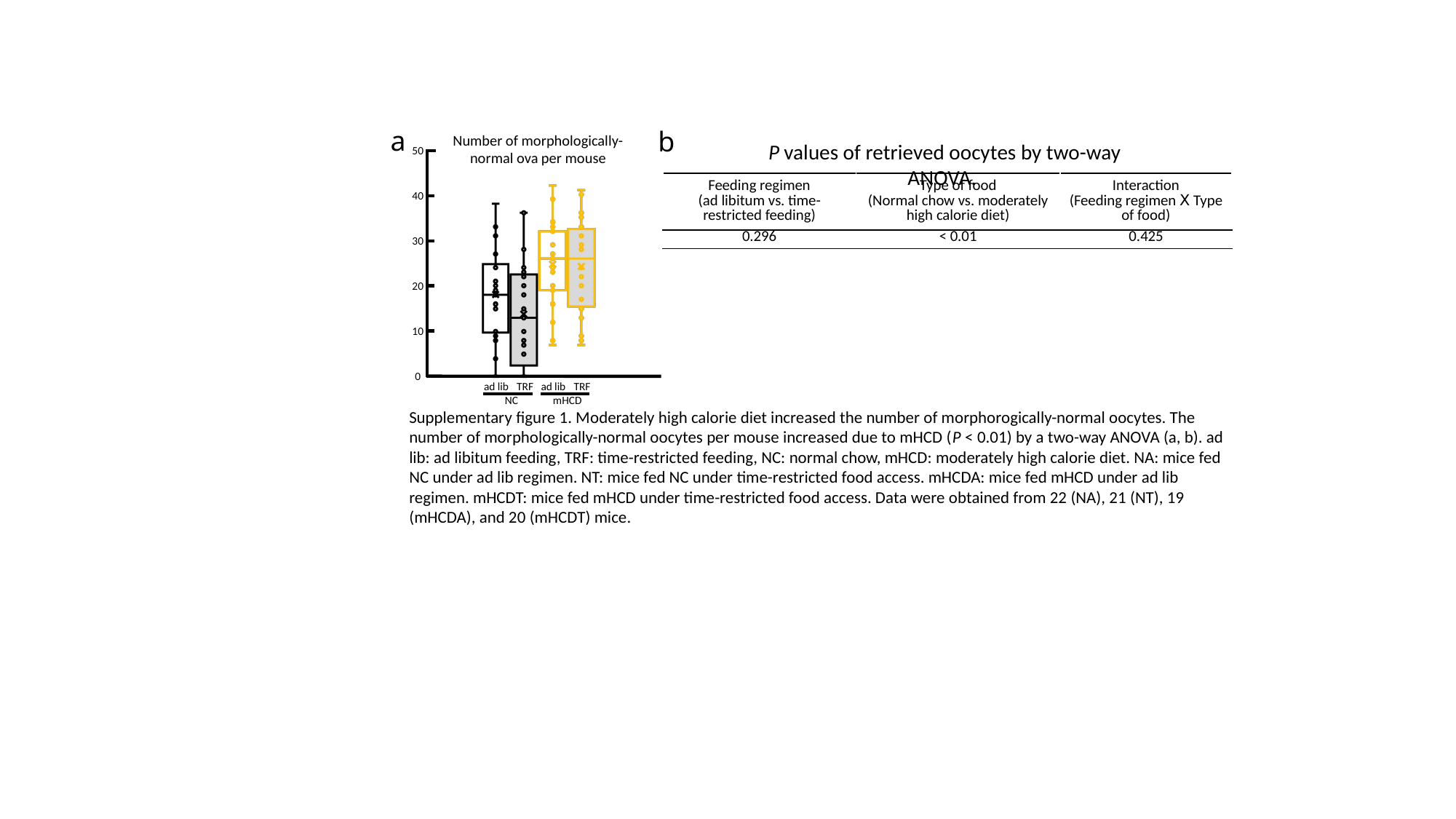

a
b
 P values of retrieved oocytes by two-way ANOVA.
| Feeding regimen (ad libitum vs. time-restricted feeding) | Type of food (Normal chow vs. moderately high calorie diet) | Interaction (Feeding regimen ꓫ Type of food) |
| --- | --- | --- |
| 0.296 | < 0.01 | 0.425 |
Supplementary figure 1. Moderately high calorie diet increased the number of morphorogically-normal oocytes. The number of morphologically-normal oocytes per mouse increased due to mHCD (P < 0.01) by a two-way ANOVA (a, b). ad lib: ad libitum feeding, TRF: time-restricted feeding, NC: normal chow, mHCD: moderately high calorie diet. NA: mice fed NC under ad lib regimen. NT: mice fed NC under time-restricted food access. mHCDA: mice fed mHCD under ad lib regimen. mHCDT: mice fed mHCD under time-restricted food access. Data were obtained from 22 (NA), 21 (NT), 19 (mHCDA), and 20 (mHCDT) mice.

## Slide 3
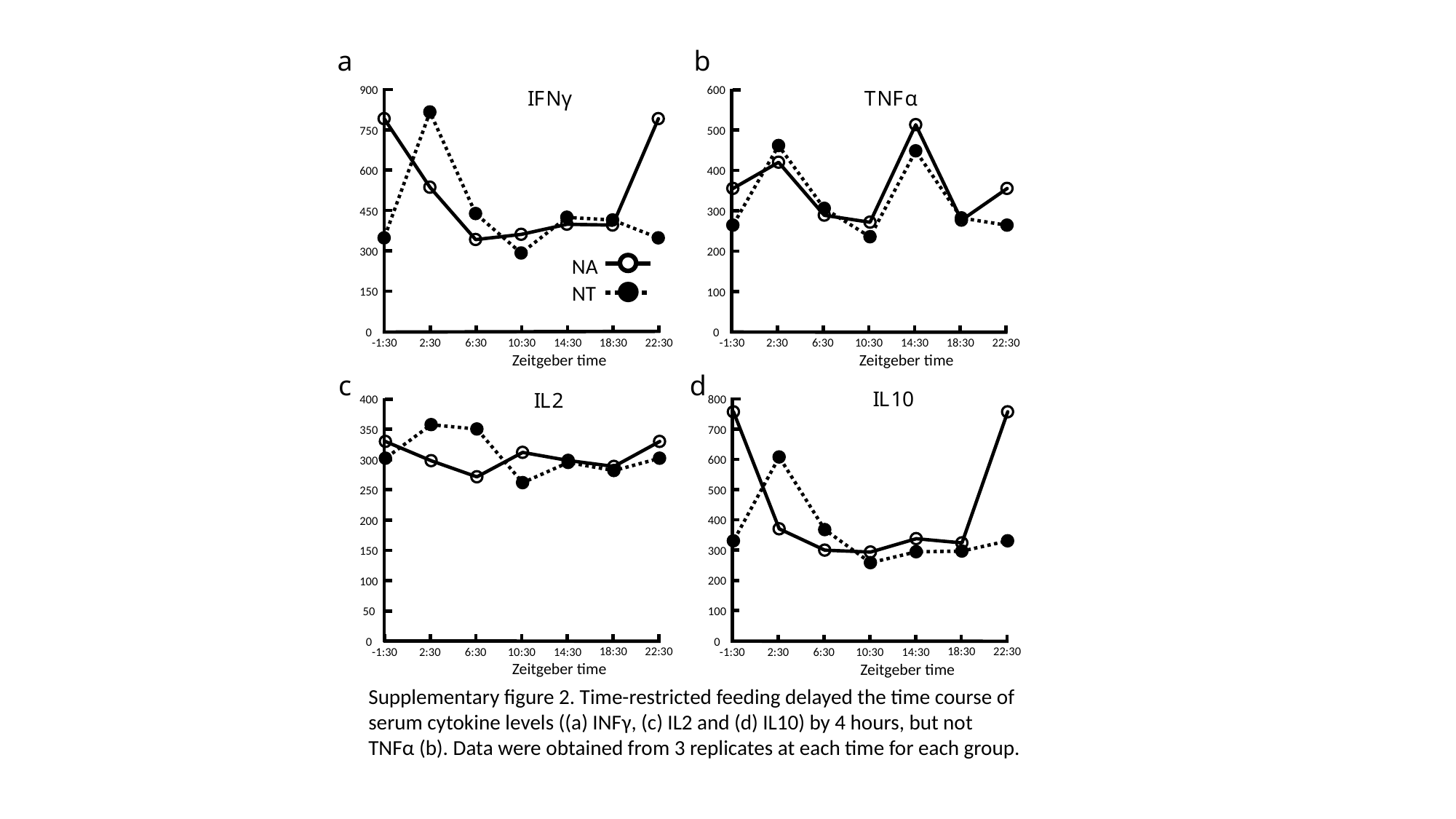

a
b
NA
NT
c
d
Supplementary figure 2. Time-restricted feeding delayed the time course of serum cytokine levels ((a) INFγ, (c) IL2 and (d) IL10) by 4 hours, but not TNFα (b). Data were obtained from 3 replicates at each time for each group.
