# Supplementary material for: Effects of time-restricted feeding and type of food on fertility competence in female mice
Source: Sci Rep. 2022 Apr 29;12:7064. doi: 10.1038/s41598-022-11251-3 (PMC9054750; doi:10.1038/s41598-022-11251-3)
Supplement: Supplementary file 3 — Supplementary Information 3. [file 41598_2022_11251_MOESM3_ESM.docx]

## Title: Effects of time-restricted feeding and type of food on fertility competence in female mice

## Authors: Nafuko Konishi, Hiroshi Matsumoto, Shu Hashimoto, Udayanga Sanath Kankanam Gamage, Daisuke Tachibana, Aisaku Fukuda, Yoshiharu Morimoto, Masayasu Koyama

**Supplementary Table 1. Weight changes over 11 weeks.**

| Group | Start | 1 week | 2 weeks | 3 weeks | 4 weeks | 5 weeks | 6 weeks | 7 weeks | 8 weeks | 9 weeks | 10 weeks | 11 weeks |
| --- | --- | --- | --- | --- | --- | --- | --- | --- | --- | --- | --- | --- |
| NA | 15.79 | 17.59^a^ | 17.9^a^ | 18.15^a^ | 19.04^a^ | 19.76^a^ | 20.18^a^ | 20.55^a^ | 20.9^a^ | 21.23^a^ | 21.42^a^ | 21.57^a^ |
| NT | 15.83 | 19.2^b^ | 18.9^bc^ | 19.52^b^ | 20.11^b^ | 20.68^b^ | 20.74^ab^ | 22.17^bc^ | 22.21^b^ | 22.62^b^ | 22.97^b^ | 22.94^b^ |
| mHCDA | 16.14 | 17.63^a^ | 18.41^ab^ | 19.24^b^ | 20.13^b^ | 20.84^b^ | 21.33^bc^ | 21.82^b^ | 22.27^b^ | 22.66^b^ | 23.14^b^ | 23.38^b^ |
| mHCDT | 16.18 | 18.78^b^ | 19.43^c^ | 20.34^c^ | 20.47^b^ | 21.18^b^ | 22.25^cd^ | 22.73^c^ | 22.88^b^ | 23.04^b^ | 23.36^b^ | 23.48^b^ |
| P values |  | < 0.01 | < 0.01 | < 0.05 | < 0.01 | < 0.05 | < 0.05 | < 0.05 | < 0.01 | < 0.01 | < 0.01 | P < 0.01 |

NA: mice fed normal chow under an ad lib regimen, NT: mice fed normal chow under time-restricted access to food, mHCDA: mice fed moderately high calorie diet (mHCD) under an ad lib regimen, HCDT: mice fed mHCD under time-restricted access to food.

^ab^Data were compared by a Tukey-Kramer test following ANOVA.

**Supplementary 3. Primer sequences for real-time RT-PCR.**

| Gene | Forward primer sequence (5’ to 3’) | Reverse primer sequence (5’ to 3’) | GeneBank |
| --- | --- | --- | --- |
| *Actin* | GGC TGT ATT CCC CTC CAT CG  (54-73) | CAG TTG GTA ACA ATG CCA TGT  (186-206) | EF095208.1 |
| *Per 2* | TGA TCG AGA CGC CTG TGC TCG T  (1,250-1,271) | CTC CAC GGG TTG ATG AAG CTG G  (1,412-1,433) | NM_011066.3 |
| *Reverb alpha* | CTG CAG GCT GAT TCT TCA CA  (372-391) | TCT TGG GGT GGC TAT ACT GC  (462-481) | NM_145434.4 |
| *Bax* | AAG CTG AGC GAG TGT CTC CGG CG  (295-317) | GCC ACA AAG ATG GTC ACT GTC TGC C  (632-656) | NM_007527.3 |
| *Bcl2* | CTC GTC GCT ACC GTC GTG ACT TCG  (1,715-1,738) | CAG ATG CCG GTT CAG GTA CTC AGT C  (1,932-1,956) | NM_009741.5 |
| *Cpt1* | AAA GAT CAA TCG GAC CCT AGA CA  (342-364) | CAG CGA GTA GCG CAT AGT CA  (446-465) | NM_013495.2 |
| *Fas* | GCT GCG GAA ACT TCA GGA AAT  (7,058-7,078) | AGA GAC GTG TCA CTC CTG GAC TT  (7,119-7,141) | NM_007988.3 |
|  |  |  |  |

**Supplementary Table 4. Standard DNA sequences for real-time RT-PCR.**

| Gene | Sequence (5’ to 3’) | GeneBank |
| --- | --- | --- |
| *Actin* | (2-210) | EF095208.1 |
| *Per 2* | (1,191-1,470) | NM_011066.3 |
| *Reverb alpha* | (351-490) | NM_145434.4 |
