# Supplementary material for: Effects of time-restricted feeding and type of food on fertility competence in female mice
Source: Sci Rep. 2022 Apr 29;12:7064. doi: 10.1038/s41598-022-11251-3 (PMC9054750; doi:10.1038/s41598-022-11251-3)
Supplement: Supplementary file 4 — Supplementary Information 4. [file 41598_2022_11251_MOESM4_ESM.docx]

## Title: Effects of time-restricted feeding and type of food on fertility competence in female mice

## Authors: Nafuko Konishi, Hiroshi Matsumoto, Shu Hashimoto, Udayanga Sanath Kankanam Gamage, Daisuke Tachibana, Aisaku Fukuda, Yoshiharu Morimoto, Masayasu Koyama

## Transcriptome analyses

The quality and concentrations of RNA was examined by Eppendorf BioPhotometer® D30 equipped with μCuvette G 1.0 (Eppendorf AG, Hamburg, Germany). Samples were obtained from 3 trials of each of the 4 groups.

The transcriptome analysis was performed using RNA obtained from the ovaries. Quality was checked using a 4200 TapeStation (Agilent Technologies, Santa Clara, CA, USA) with RNA Screen Tape. After quality check, cDNA library was prepared using the TruSeq Stranded mRNA LT Sample Prep Kit (Illumina, San Diego, CA, USA). Derived cDNA libraries were checked for quality and quantity using a4200 TapeStation with D1000 Screen Tape. Quantitative PCR was also conducted to examine the quantity of the libraries using KAPA Library Quantification kit (KAPA Biosystems, Wilmington, MA, USA). Cluster generation and sequencing was performed on a NovaSeq 6000 System as 100-bp paired end reads. The bcl2fastq2-v2.20.0 software was used for image analysis, base calling, and quality filtering following the manufacturer's instructions. We preprocessed the raw reads from the sequencer to remove low quality and adapter sequence before analysis and aligned the processed reads to the Mus musculus (mm10) using HISAT v2.1.0 ^64^. HISAT utilizes two types of indexes for alignment (a global, whole-genome index and tens of thousands of small local indices). The indexes for these two types were constructed using the same BWT (Burrows–Wheeler transform)/ a graph FM index (GFM) as Bowtie2. Due to its use of these efficient data structures and algorithms, HISAT generates spliced alignments several times faster than the widely used Bowtie and BWA. The reference genome sequence of Mus musculus (mm10) and annotation data were downloaded from the NCBI. Then, transcript assembly of known transcripts was processed by StringTie 1.3.4d ^65, 66^. Based on the results obtained, expression abundance of transcript and gene were calculated as read count or FPKM value (Fragments Per Kilobase of exon per Million fragments mapped) per sample. The expression profiles are used to do the additional analysis such as DEG (Differentially Expressed Genes). In groups with different conditions, differentially expressed genes or transcripts can be filtered by statistical hypothesis testing.

## Statistical analysis of gene expression level

The relative abundance of genes was measured in FPKM using StringTie. Statistical analysis was performed to find differentially expressed genes using the estimates of abundance for each gene in samples. Those genes with 0 FPKM value in at least one sample were excluded. To facilitate log2 transformation, 1 was added to each FPKM value of filtered genes. Filtered data were log2-transformed and subjected to quantile normalization. The statistical significance of the differential expression data was determined using independent t-test and fold change in which the null hypothesis was that no difference exists among the 4 groups. The false discovery rate (FDR) was controlled by adjusting the *P* value using the Benjamini-Hochberg algorithm. For the DEG set, hierarchical clustering analysis was performed using complete linkage and Euclidean distance as a measure of similarity. The gene-enrichment analysis and KEGG pathway analysis for DEGs were also performed based on Gene Ontology (http://geneontology.org/) and KEGG pathway (https://www.genome.jp/kegg/) database respectively.

## Hierarchical clustering

Hierarchical clustering analysis was also performed using complete linkage and Euclidean distance as a measure of similarity to display the expression patterns of differentially expressed transcripts which are satisfied with |fold change|≥1.5 and raw *P* <0.05.

All data analysis and visualization of differentially expressed genes was conducted using R 3.6.0 software (www.r-project.org).

The expression levels of genes of interest related to the immune system (complement and coagulation cascades and natural killer cell mediated cytotoxicity), endocrine system (PPAR signaling pathway), digestive system (cholesterol metabolism), immune diseases (systemic lupus erythematosus, rheumatoid arthritis), and infectious diseases were compared among the 4 groups. Information on target genes involved in mice was obtained from the Kyoto Encyclopedia of Genes and Genomes database (map No. 0610, 04650, 03320, 04979, 05322, 05323).

1. Kim E, Hwang S, Kim H, Shim H, Kang B, Yang S, Shim JH, Shin SY, Marcotte EM, Lee I. MouseNet v2: a database of gene networks for studying the laboratory mouse and eight other model vertebrates. *Nucleic Acids Res.* 2016; 44:D848–854.
2. Pertea M, Pertea GM, Antonescu CM, Chang TC, Mendell JT, Salzberg SL. StringTie enables improved reconstruction of a transcriptome from RNA-seq reads. *Nat Biotechnol.* 2015; 33:290–295.
3. Pertea M, Kim D, Pertea GM, Leek JT, Salzberg SL. Transcript-level expression analysis of RNA-seq experiments with HISAT, StringTie and Ballgown. *Nat Protoc.* 2016; 11:1650–1567.
